# What do women value in a maternal education app? A sequential mixed-methods study on user perspectives of EMAeHealth

**DOI:** 10.18332/ejm/223919

**Published:** 2026-06-29

**Authors:** Maite Espinosa, Isabel Artieta-Pinedo, Carmen Paz-Pascual, Ema-Q Group, Itziar Estalella, Amaia Maquibar

**Affiliations:** 1 Osakidetza-Basque Health Service, Bilbao, Spain; 2 Biobizkaia Health Research Institute, Barakaldo, Spain; 3 University of the Basque Country (EHU), Leioa, Spain

**Keywords:** mHealth app, pregnancy, antenatal care, digital health apps

## Abstract

**INTRODUCTION:**

eHealth is a resource that provides continuity of care from midwives and maternal education, allowing for the personalization of information and the selection of resources tailored to individual needs. However, for many available digital tools, information on their quality and usability is lacking. The objective of this study was to explore the perceptions of users of the EMAeHealth digital app, which was designed and developed by midwives, regarding its acceptance, usability, strengths, and weaknesses for implementation.

**METHODS:**

This was an exploratory sequential mixed-methods study. Semi-structured individual interviews were conducted between January and March 2024. Participants were selected by purposive sampling. Subsequently, an ad hoc survey was created based on these results to be filled in anonymously.

**RESULTS:**

The acceptance rate of the EMAeHealth app was 64%. In the qualitative analysis, there were two categories: 1) ‘What makes this app stand out?’, including accessibility, quantity, quality, good organization of information, and credibility of the source; 2) ‘Remaining potential’, describes the improvement recommendations that were most strongly agreed with, both in the interviews and in the survey with 106 women, which were related to personalization, inclusion of a chat box with the midwife, and connection of the app with the health records services, with ratings 4.38 ± 0.83, 4.28 ± 1.00 and 4.16 ± 1.10 out of 5 points, respectively.

**CONCLUSIONS:**

A digital tool would expand the availability of information, personalization, and resources offered in maternal education, increasing its reach and effectiveness. However, it is necessary to carry out further work on the individualization of information and its adaptation to each woman’s health situation and stage of life.

## INTRODUCTION

eHealth can help health-service users take a more active role in decision-making and help health professionals guide the patient in this process^1^. It can also contribute, at the organizational level, to a more patient-centered model of healthcare^2^. The World Health Organization has highlighted the relevance of digital health interventions to address health needs, although they stress that these tools should always be used as an aid and improvement for health systems, not as a substitute^3^. Currently, most digital healthcare tools (eHealth) are accessible from mobile devices such as smartphones or tablets (mHealth).

Pregnancy and postpartum are periods of great vulnerability in which sensitivity to information and self-care is heightened. Midwives respond to this need with woman-centered health education programs, focusing not only on pregnancy care but also on women’s well-being and satisfaction, providing information, emotional support, and resources to cope with childbirth and parenting both individually and in groups^4^. However, access to this information and resources is limited to appointment times or group activities, while women’s needs occur continuously, with unexpected variations. Digital technology can increase the reach and effectiveness of this work by providing continuous, immediate, and personalized support^5,6^. Indeed, young women from differing socioeconomic backgrounds consistently use digital content as a source of information about their health, and the information they consult is positively correlated with the decisions they make about their health^7^. However, according to the WHO, the enthusiasm for digital health has also driven a proliferation and overwhelming diversity of digital tools, although there is still limited understanding of their impact on health systems and people’s well-being^3^. Some mHealth apps are not evidence-based, prioritize diseasecentered over person-centered approaches, have limited usability, and present serious concerns regarding user privacy^3,5,7,8^. According to the WHO, reviewing the needs of health systems and moderating expectations based on Information and Communication Technologies (ICTs) is of considerable value^3^.

The EMAeHealth (eHealth Maternal Education) application was designed as a complement to Maternal Education (ME) and is organized into four areas: information, communication, self-management of health, and clinical data^9^. Although the information area is freely accessible, entry into the self-management and clinical data areas requires authentication by the user. It allows for the assessment of perinatal psychosocial needs and promotes self-management of health and well-being in the postnatal period, proposing plans to change eating habits, exercise, behavior, and decisions. The app is being studied, both in terms of implementation and clinical efficacy, using a hybrid efficacy-implementation design^9,10^.

The key factors for the successful implementation of e-health applications are usability^11^, technological performance, and user participation^3^. However, few digital health apps publish the results of their usability assessment^11^. Usability is generally measured in terms of aspects such as user ratings of the application’s flexibility, operability, comprehensibility, ease of learning, efficiency, satisfaction, attractiveness, consistency, and error rates^12^. In recent years, numerous scales have emerged to measure the quality of health apps, quantitatively assessing areas such as engagement, functionality, aesthetics, information quality, potential for behavioral change^11,12^, and providing an overall measure of usability. However, they do not identify the user perceptions of the tool’s effectiveness, their satisfaction, their preferences, or the shortcomings they identify, problems that need to be addressed; in this sense, qualitative methods may be more useful^3,11^.

The aim of this study is to explore users’ perceptions of the EMAeHealth digital app about its acceptance, usability, strengths, and weaknesses for its implementation in real-world conditions.

## METHODS

### Design

This is an exploratory sequential mixed-methods research study^13^. This study design was chosen for its appropriateness in the research of health service interventions, as the qualitative methods allow for the exploration of unexpected issues while the quantitative methods address the limited generalizability of the former. In this study, both methods were used with a developmental perspective, in the sense that qualitative results informed the content of the questionnaire used for quantitative data collection. Initially, the qualitative study was carried out to discover whether an app like this could offer any advantages to already existing in-person antenatal education programs and other pregnancy apps. After this qualitative study, the quantitative study was designed based on the main findings of the qualitative study, to assess the agreement of a greater sample of users with the experiences reported by participants in the qualitative study in relation to app use and potential improvements^13^.

### Participants

This study is part of a comprehensive research project in which the perceptions and needs of women during pregnancy, childbirth and postpartum have been analyzed, as well as the resources available to them to adapt to each moment of the process^9^. Eligible participants for this study were all pregnant Spanish-speaking women who had seen their midwives at the start of gestation and for 1 year after the implementation of the EMAeHealth app. For the interviews, 39 women who had used it were invited by phone following a purposive sampling. Saturation was reached after the tenth interview. The subsequent survey was launched online to 400 women who had been part of the study, collecting responses anonymously.

### Data collection

The qualitative results were collected through individual semi-structured interviews, recorded, and then professionally transcribed. Data collection took place between January and March 2024 using individual semi-structured interviews. All the participating women received an information sheet about the study beforehand and signed an informed consent form. Verbal consent was also obtained for recording the interviews, which were conducted via video call. The mean duration of the interviews was about 16 minutes, with a range 13–28 minutes. The interview guide included questions related to the use of the app, some aspects of usability, comparison with other apps for pregnancy, and recommendations for improvement.

The quantitative results were collected in December 2024 through an anonymous online survey created ad hoc, based on the results of the qualitative study and therefore including questions that were part of aspects raised by the participants in the interviews. A structured questionnaire was designed, with a filter question to divide the respondents into those who had used the app and those who had not, and who were asked for the reasons for not using it. For those who had used it, the questionnaire consisted of 7 items, including Likert scale questions (1=strongly disagree to 5=strongly agree) and closed-ended questions, covering aspects of usability, along with questions to assess agreement with the improvement recommendations given by participants in the qualitative study. All the women who had activated the tool were invited to answer the questionnaire, without differentiating between pregnant women and women who had already given birth.

### Data analysis

The transcriptions were analyzed following qualitative content analysis as described by Graneheim et al.^14^ with the support of Atlas.ti software for the coding process and the retrieval of quotations. AM and IE read all the transcriptions many times to familiarize themselves with the data, and each interview was independently double-coded by each of them to capture all the relevant information. The coding process was deductive, as identified and coded meaning units were interview extracts that were related to the questions stated in the objective, i.e. acceptance, usability, strengths, and weaknesses of the app being evaluated. The same two authors who drew up the codes carried out a preliminary sorting of codes into categories that encompassed the core meaning of the codes included in each of them. This preliminary sorting was later discussed by all the authors and modified after agreements were reached until the final version of the results described in the following section was approved by all the authors.

Some measures were implemented in this study to enhance trustworthiness. First, to achieve credibility, a relationship of trust was established with the participants by providing detailed information about data management and the measures taken to ensure confidentiality. The accuracy of the interview transcripts was ensured by hiring a professional service for this purpose, and the authors who did the coding double-checked the accuracy of the transcriptions by listening to the audios. Triangulation of the results by the five researchers improved credibility. Three of the researchers had an insider perspective of the app being assessed as they had been part of the development and testing process from the beginning, while the other two authors were not directly involved in the whole process. These different positions enriched the credibility by combining these two different perspectives. In terms of confirmability, representative quotations from participants have been included in the Results section. Furthermore, in terms of transferability, participants’ relevant characteristics have been summarized to facilitate readers’ assessment of the possible application of these results to other settings/ participants.

The analysis of the quantitative data was carried out using descriptive statistical methods, i.e. frequency and percentage, and mean with standard deviation (SD) for Likert scale responses using SPSS for Windows version 26.

## RESULTS

The acceptance rate of the EMAeHealth digital app was 64%. The characteristics of the women who used the EMAeHealth app (n=254) and interviewees (n=10) are presented in Table 1. For the qualitative study, 10 interviews were carried out. In the quantitative section, 106 out of 400 women responded to the survey (response rate: 26.5%).

**Table 1:** Sociodemographic characteristics of women who used the EMAeHealth app (N=254) and interviewees (N=10) in the mixed-methods study conducted in the Basque Country, Spain, January–December 2024

*Characteristics*	*Women who* *used the* *EMAeHealth* *app* *n (%)*	*Women who* *responded to* *the interviews* *n (%)*
**Age** (years), mean (SD)	32.8 (3.2)	33.0 (3.4)
**Studies**		
Non- University	87 (34)	5 (50)
University	167 (66)	5 (50)
**Nationality**		
Spanish	236 (93)	10 (100)
Other	18 (7)	
**Work**		
Paid	227 (89)	10 (100)
Housewife	27 (11)	
**Number of children**		
0	170 (67)	5 (50)
1	72 (28)	3 (30)
2	12 (5)	2 (20)

### Qualitative results

Two categories were created from the qualitative analysis. The first, ‘What makes this app stand out’, compiles all the strengths participants saw in this app, both in comparison to in-person antenatal health education programs and in relation to other available apps. The second, ‘remaining potential’, describes potential improvements that would make the app better and enhance its use.

*What makes this app stand out*

Interviewees overall assessed the app very positively. They considered it very intuitive and easy to manage and appreciated the inclusion of questionnaires about nutrition, physical activity, and emotional status. Accessibility, especially compared to in-person health education sessions that require asking for leave from work or, in some cases, the need to change work shifts, was one of the main benefits highlighted:

*‘I think having it somewhere online is more convenient than a talk or something, because … You can look at it whenever you want, without having to miss work or change shifts.’* (P2)

Access to the app required the use of a digital certificate that is commonly used for access to different public administration services in the region. As reflected in the following quotation, although this requirement was generally considered an easy identification system, some participants mentioned that it might be a barrier for some women to use the app:

*‘… I don’t know, it’s easier to use your fingerprint to let you access it, or a simple code.’* (P1)

In relation to more specific features, quantity, quality, and good organization of the information, referring to the ease of finding specific information overall, and related to the different stages of the pregnancy were among the reasons for the positive valuation. More specifically, the credibility of the source of the information – healthcare professionals – was a remarkable strength of this app in comparison to other sources of information:

*‘I really did like it because it is sort of organized in stages. When you want to get pregnant, then when you get pregnant, the birth, the postpartum, then the issue of nutrition … so I think it’s good* … *in fact, it is well explained.’* (P8)

*‘You have so much information in so many places that you don’t know which one is the right one, for sure, and this is a way in the end, since there are professionals behind it, and you can see that this is the right one.’* (P9)

Finally, in relation to the content of the information provided, one participant highlighted the non-judgmental approach when providing information about the different options when feeding a newborn as very positive, and commented on how this respectful approach helped her feel supported:

*‘It’s not like in other places where they present formula milk as something horrible and, ugh, what are you doing* … *You are going to kill your child. Here, I mean in the app, I did not feel that way, so, from that point of view, I am grateful because in the end, it’s a decision that I made that was difficult, but I decided it, and I would like to feel supported. With the app, I felt supported.’* (P8)

It is worth noting that there were some differences in the usefulness perceived depending on whether the current pregnancy was the first or subsequent, and it was more highly valued by those in their first pregnancy. Similarly, those in the first pregnancy stated they had made more use of the app than those in subsequent pregnancies and were eager to have more information. For those women who were pregnant while taking care of other children, a lack of time was a repeated barrier to making use of this or other apps:

*‘It would have been useful in the first pregnancy, as in the end, in the second [pregnancy], it seems that you’ve already lived through the experience, so you don’t look at it as much.’* (P2)

*Remaining potential*

The main potential improvement suggested for the app was linked to its origin and ownership being part of the regional public healthcare system. Therefore, participants considered that the app had the potential to become essential if new functions related to healthcare provision were added by linking it to individual health records. Participants believed a huge difference with other existing pregnancy apps would be the incorporation into the app of all the tests each woman had to undergo during the pregnancy, with the date, reminders of the appointment, aim of the test, explanation of the procedure, results, and implications of the results:

*‘Maybe it would be good to have more explanations when it comes to what type of tests they are going to do on me or what they consist of, because really, you haven’t a clue in the end.’* (P10)

The second main recommendation arose from other apps women knew and was related to making the app more individualized, for example by including notifications for each week of the pregnancy, providing information about the development of the baby in that week, healthy lifestyle recommendations for that specific moment, and individualized support and guidance for the results from the questionnaires in the app about nutrition, physical activity and emotional status:

*‘[The app] could include the week you have reached, a bit of the initial information, and every week you could get a notification of a weekly news item, how your body is changing, how the baby is growing, all those things* … *And if you add it to a plan that you already have, well, in the end, it is quite complementary.’* (P4)

In any case, participants perceived that none of the possible improvements would completely counterbalance the lack of opportunity to share experiences with other women in the same situation and to develop networks that can offer support and companionship throughout the pregnancy and after the birth that face-to-face antenatal education provides. Yet, they believed this could be mitigated to some extent by having something similar to a chat box in the app, managed by a professional who knows who you are, where specific doubts would be answered, even if not at the moment, during the day, for example:

*‘If you have a question or you are feeling down or you have a doubt, there should be a kind of chat box, someone who is on the other screen, who reads your messages, not on the same screen, but well, when they can, but they know that you are Mary, that you are 30, that you are pregnant, that you are in such-and-such a week and they know a little about who you are.’* (P3)

Finally, in comparison with other popular apps for pregnancy that participants were using, the improvement recommendations included adding some information for the partners of pregnant women, making the app more visually attractive by adding more images related to the information given, and taking into account different pregnancies, like multiple or *in vitro* fertilization processes:

*‘Images of babies, of their size, weight* … *what it’s like inside the amniotic fluid; you see all of that in other apps, and that’s missing here, that’s what I think it lacks.’* (P7)

*‘Or, for example, it [another app] also differentiates if it’s a twin birth because, in the end, that is also different, and it has a little section for the partner, so that they know what’s happening week by week.’* (P4)

### Quantitative results

In relation to the survey, of the 106 women surveyed, 64 used the app. The main reasons for not using the app were difficulty of access, lack of time, and already using a better-liked app.

In terms of usability, the app’s aesthetics were rated as 3.70 out of 5. The majority of respondents would add more content, i.e. sections, functions, and/or questionnaires for the self-management of health, but overall, they think that the current length of the topics is fine. Those who had filled in a questionnaire for the self-management of health (86%) found it useful to reflect or make changes (rated 3.33/5), and for those who had decided to start a change plan (80%), the app was useful for them in the process (rated 2.79/5).

Finally, the improvement recommendations given by those interviewed that had the strongest agreement were: ‘That it could be more personalized (weeks of gestation, changes made …)’, ‘Include a chat box to resolve doubts with my midwife’ and ‘Connect the app to the official Health File to see appointments, test results, etc.’ with ratings of 4.38 ± 0.83, 4.28 ± 1.00 and 4.16 ± 1.10 out of 5 points, respectively (Table 2).

**Table 2:** Results of the ad hoc questionnaire from the mixed-methods study conducted in the Basque Country, Spain, January-December 2024, in relation to the EMAeHEALTH app use (n=106) and its usability, aesthetics and improvement suggestions according to users’ experience (n=64)

*Items*	*n (%)*
**Have you attended any face-to-face antenatal education classes?**	
Yes	82 (77.4)
No	24 (22.6)
**Have you been able to use the EMA eHealth app?**	
Yes	64 (60.4)
No	42 (39.6)
***Unable to use the app* (N=46)**	***n***
**Could you tell us why you did not use the EMA eHealth app?**	
Lack of time	10
Difficulty of access	11
I didn't think it was necessary	8
I had another app that I liked better	9
Other	8
***Able to use the app* (N=64)**	***Mean ± SD***
**Rate the following aspects of the EMA eHealth app** (1=difficult to 5=easy)	
App download	4.19 ± 0.89
access once downloaded	4.08 ± 0.99
browsing the app	3.91 ± 1.02
Finding the information	3.69 ± 1.07
**How did you find the app visually?** (1=displeasing to 5=pleasing)	3.70 ± 0.77
	***n (%)***
**Does the content seem appropriate to you?**	
No, I would like the app to have more sections or functions	14 (21.9)
No, I would like more topics or more questionnaires for self-management of health	21 (32.8)
Yes, I found the content appropriate	29 (45.3)
**Do you think the length of each topic is appropriate?**	
No, I think they are generally very long	4 (6.3)
No, the topics are covered too briefly	15 (23.4)
Yes, in general the length of the topics is good	45 (70.3)
	***Mean ± SD***
**If you have answered any questionnaire for the self-management of health, did you find it useful to reflect on or make any changes?** (N=55) (1=not useful to 5=very useful)	3.33 ± 0.94
**If you have decided to start a change plan (diet, exercise, emotional or behavioral issue), was the app useful to you in the process?** (N=51) (1=not useful to 5=very useful)	2.79 ± 1.08
**What grade would you give the app in general?** (low=1 to high=5)	3.63 ± 0.81
**Rate the following possible improvements to the app** (1=not useful to 5=very useful)	
Include a forum that allows me to share experiences with other pregnant women	4.13 ± 0.90
Include a chat box to resolve doubts with my midwife	4.28 ± 1.00
Connect the app to the official Health File to see appointments, test results, etc.	4.16 ± 1.10
Make it more personalized (e.g. weeks of gestation, changes made)	4.38 ± 0.83
Receive notifications for weeks of gestation with information about that week	4.05 ± 1.13
Increase visual content	3.90 ± 1.02

## DISCUSSION

Pregnant women belong to a group that is particularly willing to seek information and advice at this stage^15-17^. In our study, 64% of women use EMAeHealth, a slightly lower percentage than that seen in other studies, 75%^17^ or 77%^16^. In our healthcare system, government applications are less widespread than in other countries in Northern Europe^18,19^. One possible interpretation for this difference is the reluctance of healthcare professionals who perhaps do not feel sufficiently confident about the information and security offered by these tools and do not recommend them or recommend them with reservations^18,20^.

The results of this study show that women, especially first-time mothers, appreciate the immediacy and availability of this digital tool in their daily lives, its clear and non-judgmental presentation, and the credibility lent by having been developed by midwives. However, they would like the information to be fully personalized, based on gestational age, the progress of their pregnancy, and their responses to questionnaires about diet and exercise. They also miss peer interaction through the app or, at least, direct contact with a midwife. These impressions are confirmed by the quantitative study, which rated the content and format of the app’s information section at 3.7/5. However, in the questions about completing questionnaires and self-managing health, criticism already expressed in the interviews regarding the lack of personalized responses reappeared. Although they found the questionnaires useful and rated them 3.3/5, these surveys did not use them to help change behavior, such as diet or exercise (2.79/5). Undoubtedly, behavioral change is affected by factors that go beyond knowledge or even the person’s motivation, such as the social context^4^. From this perspective, the application could help raise awareness about possible change, but closer intervention and follow-up would be needed to modify behavior. In line with the interviews, the survey of more than 100 women confirmed the demand for personalized responses (4.38/5) and peer interaction (4.28/5). The results are consistent with other studies conducted in similar settings^16-18,21^. Women who used the app particularly valued the permanent accessibility of information^15^ and the credibility that comes with being hosted by a public health system^20^. On commercial websites and apps related to pregnancy, the variability of information and lack of accuracy cause women anxiety and frustration^16,22,23^.

The EMAeHealth app scored highly in terms of ease of download and access, but it was difficult to locate the desired information. The content was considered reasonably good, although 22% thought it should have more features and perhaps some topics aimed at partners. These drawbacks may justify less use of the resources offered by the app (such as starting a plan to change diet or exercise). Some studies show that minimal technical difficulties determine both the acquisition and use of an app^16,21,22^. The intuitive use of the application is also a determining factor for participation, especially when the user is accustomed to the aesthetics of social media^22,24,25^. In our opinion, health authorities can do little to compete with the power of large commercial or technology companies, which are capable of continuously improving the design and interoperability of digital tools^26^. Commercial applications offer automated, intuitive features, and very simple language complemented by graphics and gamification^17,18,27^. The public system can and should strive to offer some of these resources, but there will always be delays and limitations caused by control systems. Fortunately, this does not mean that their influence on health behavior will necessarily be less. In a study in which 50 young adults were asked about the importance they attached to online health information, Briones et al.^28^ found that participants were critical of the online medium, using it for entertainment, as a means of finding contacts, or as a source of health information, depending on the credibility they attributed to it.

The protection of privacy and the implementation of barriers that prevent strangers from accessing clinical data are mandatory requirements in the public health system that women perceive as a difficulty^19,21^, but which are also highly valued by users^15^. This tension between the need for privacy and the importance of receiving information relevant to each individual constitutes the ‘personalization–privacy paradox’ and, as Cloarec et al.^29^ show, it is frequently resolved with a preference for personalization over privacy when the information is perceived to be of interest. Specifically, in our study, women felt that the app should use their relationship with individual clinical data to offer personalized information, appointments, notifications, and self-care advice tailored to their stage of pregnancy or specific condition. The lack of alignment between what the tool offers and women’s specific needs causes disengagement^23^, while reminder messages and information tailored to the personal situation improved engagement with the digital tool^24,25^.

In line with previous studies, women perceived a lack of personal connection with professionals and support groups^17,25^. Although the use of apps improved self-care in relation to weight, blood pressure, or blood glucose^30^, they preferred to have a ‘real’ point of contact during pregnancy or postpartum^21,23^. A mixed approach, both online and in person, has proven to be effective and satisfactory during pregnancy^17,23^. One difficulty the healthcare system faces in meeting this demand is that managing a peer social network requires sustained dedication and technical knowledge on the part of professionals, who often do not have the resources to moderate this environment^18,20^.

Despite this, the use of new technologies in women’s healthcare allows midwives to advance in providing care focused on women’s experiential needs, not just clinical ones^31^. Having continuous and personalized attention fosters reflection, recognition, and expression of women’s preferences and personal desires^32,33^. A digital tool would make it possible to adapt prenatal education to their needs, develop a birth plan according to their own wishes, and experience childbirth and parenting in a way that goes beyond what is prescribed by the medical services^33^.

The sociodemographic profile of women who have used the tool is also reflected in their use of other health services^34^. Most of these women have a medium-high level of education and live in an urban environment^16,17,35^. Among other factors, this selection may be related to difficulties in using the tool or understanding the language^21^. In addition, identification is required, which can only be obtained if certain requirements are met, thereby discriminating against non-native or unregistered groups^21^. Despite their potential beneficial effect in improving self-care among most citizens, these technologies may increase inequalities in the use of health services and undermine the universality of public health services^26,35^.

### Limitations

The response rate to the questionnaire was low, and the number of women who decided to participate in the interviews was small. Women who decided to participate in the study might have been more motivated to use the app and value it more positively than those who decided not to participate, with the risk of the results being weakened by non-response bias. Even though the use of a mixed methods approach might have partially overcome this limitation by the complementary perspective provided by each research method, repeated cross-sectional studies using validated questionnaires in the coming years are necessary to increase the soundness of current findings. Furthermore, the representation of women in the population with a foreign background who used the app is limited. Similarly, no difference was examined between pregnant and postpartum women in their responses to the quantitative study. It is possible that there was a recall bias related to the time elapsed since the tool was used. Therefore, future studies in our context should address this issue by focusing specifically on this population and their needs in relation to maternal education to assess if they are being met.

## CONCLUSIONS

Women value the accessibility and reliability offered by a digital tool from the public healthcare system. It is not easy for this tool to compete with other commercial tools in terms of aesthetics, gamification, etc., as it must prioritize aspects such as privacy, which are not required by the former. However, areas for improvement have been identified, such as combining digital intervention with face-to-face care, thereby reinforcing confidence in the application, or personalizing messages according to the circumstances or characteristics of each woman. This individualization, including language options, would prevent the exclusion of the most vulnerable people. The evaluation of the EMAeHealth app using a mixed-methods approach allowed us to explore which features and functionalities are most valued and subsequently confirm that this assessment is shared by a broad population. Tools like the one presented in this study offer continuous and more specifically targeted support for each woman, and encourage women to express their needs, select resources, and adapt them to their preferences, thus promoting their own healthcare. The participation of professionals would improve the effectiveness of these tools.

## Data Availability

The data supporting this research are available from the authors on reasonable request.

## References

[1] Ruiz-Azarola A, Perestelo-Pérez L. Participación ciudadana en salud: formación y toma de decisiones compartida. Informe SESPAS 2012 [Citizens’ participation in health: education and shared decision-making. SESPAS Report 2012]. Gac Sanit. 2012;26 Suppl 1:158-161. doi:10.1016/j.gaceta.2011.10.005 22265652

[2] Kalra D, Artmann J, Stroetmann V, Boye N. The eHealth contribution to person-centred care. In: Rosenmöller M, Whitehouse D, Wilson P, eds. Managing eHealth: from vision to reality. Palgrave Macmillan; 2014:211-223. 10.1057/9781137379443_14

[3] World Health Organization. WHO guideline: recommendations on digital interventions for jhealth system strengthening. World; 2019. Accessed June 1, 2026. https://iris.who.int/server/api/core/bitstreams/c3c53f30-23cc-48d0-a3b5-c05ddd7f5349/content 31162915

[4] Lopes MI, Vieira M, Cardoso A. Women’s empowerment for active labor: a qualitative study with nurse-midwives in antenatal education for childbirth. Eur J Midwifery. 2024;8:10.18332/ejm/188117. doi:10.18332/ejm/188117 PMC11339881 39175493

[5] Rockliffe L, Peters S, Heazell AEP, Smith DM. Understanding pregnancy as a teachable moment for behaviour change: a comparison of the COM-B and teachable moments models. Health Psychol Behav Med. 2021;10(1):41-59. doi:10.1080/21642850.2021.2014851 34993005 PMC8725882

[6] Vickery M, van Teijlingen E, Hundley V, Smith GB, Way S, Westwood G. Midwives’ views towards women using mHealth and eHealth to self-monitor their pregnancy: a systematic review of the literature. Eur J Midwifery. 2020;4:36. doi:10.18332/ejm/126625 33537637 PMC7839093

[7] Gjestvang C, Haakstad LAH. Navigating pregnancy: information sources and lifestyle behavior choices-A narrative review. J Pregnancy. 2024;2024:4040825. doi:10.1155/2024/4040825 39346810 PMC11438513

[8] Frid G, Bogaert K, Chen KT. Mobile health apps for pregnant women: systematic search, evaluation, and analysis of features. J Med Internet Res. 2021;23(10):e25667. doi:10.2196/25667 34524100 PMC8561408

[9] Artieta-Pinedo I, Paz-Pascual C, Bully P, Espinosa M; EmaQ Group. Design of the maternal website EMAeHealth that supports decision-making during pregnancy and in the postpartum period: collaborative action research study. JMIR Form Res. 2021;5(8):e28855. doi:10.2196/28855 34383670 PMC8386364

[10] Espinosa Cifuentes M, Artieta-Pinedo I, Paz-Pascual C, Bully-Garay P, García-Alvarez A; ema-Q Group. EMAeHealth, a digital tool for the self-management of women’s health needs during pregnancy, childbirth and the puerperium: protocol for a hybrid effectiveness-implementation study. BMJ Open. 2022;12(9):e055031. doi:10.1136/bmjopen-2021-055031 PMC9438065 36575817

[11] Maramba I, Chatterjee A, Newman C. Methods of usability testing in the development of eHealth applications: a scoping review. Int J Med Inform. 2019;126:95-104. doi:10.1016/j.ijmedinf.2019.03.018 31029270

[12] Jake-Schoffman DE, Silfee VJ, Waring ME, et al. Methods for evaluating the content, usability, and efficacy of commercial mobile health apps. JMIR Mhealth Uhealth. 2017;5(12):e190. doi:10.2196/mhealth.8758 29254914 PMC5748471

[13] Tariq S, Woodman J. Using mixed methods in health research. JRSM Short Rep. 2013;4(6):2042533313479197. doi:10.1177/2042533313479197 23885291 PMC3697857

[14] Graneheim UH, Lindgren BM, Lundman B. Methodological challenges in qualitative content analysis: a discussion paper. Nurse Educ Today. 2017;56:29-34. doi:10.1016/j.nedt.2017.06.002 28651100

[15] Leuzzi G, Recenti F, Giardulli B, Scafoglieri A, Testa M. Exploring digital health: a qualitative study on adults’ experiences with health apps and wearables. Int J Qual Stud Health Well-being. 2025;20(1):2447096. doi:10.1080/17482631.2024.2447096 39726066 PMC11703456

[16] Lupton D, Pedersen S. An Australian survey of women’s use of pregnancy and parenting apps. Women Birth. 2016;29(4):368-375. doi:10.1016/j.wombi.2016.01.008 26874938

[17] Oelhafen S. Digital health in perinatal care: exploring usage, attitudes, and needs among Swiss women in urban and rural settings. Digit Health. 2024;10:20552076241277671. doi:10.1177/20552076241277671 39233895 PMC11372771

[18] Giebel GD, Speckemeier C, Abels C, et al. Problems and barriers related to the use of digital health applications: scoping review. J Med Internet Res. 2023;25:e43808. doi:10.2196/43808 37171838 PMC10221513

[19] Hägglund M, Kharko A, Bärkås A, et al. A Nordic perspective on patient online record access and the European Health Data Space. J Med Internet Res. 2024;26:e49084. doi:10.2196/49084 38935430 PMC11240068

[20] Dahlhausen F, Zinner M, Bieske L, Ehlers JP, Boehme P, Fehring L. Physicians’ attitudes toward prescribable mHealth apps and implications for adoption in Germany: mixed methods study. JMIR Mhealth Uhealth. 2021;9(11):e33012. doi:10.2196/33012 34817385 PMC8663495

[21] Giebel GD, Abels C, Plescher F, et al. Problems and barriers related to the use of mHealth apps from the perspective of patients: focus group and interview study. J Med Internet Res. 2024;26:e49982. doi:10.2196/49982 38652508 PMC11077409

[22] Sjöström A, Hajdarevic S, Hörnsten Å, Isaksson U. eHealth literacy and health-related internet use among Swedish primary health care visitors: cross-sectional questionnaire study. JMIR Form Res. 2024;8:e63288. doi:10.2196/63288 39637377 PMC11637456

[23] Progga FT, Rubya S. Women’s perspectives and challenges in adopting perinatal mental health technologies. Proc ACM Hum-Comput Interact. 2025;9(1):1-30. doi:10.1145/370121740909183

[24] Park DY, Kim H. Determinants of intentions to use digital mental healthcare content among university students, faculty, and staff: motivation, perceived usefulness, perceived ease of use, and parasocial interaction with AI chatbot. Sustainability. 2023;15(1):872. doi:10.3390/su15010872

[25] Amagai S, Pila S, Kaat AJ, Nowinski CJ, Gershon RC. Challenges in participant engagement and retention using mobile health apps: literature review. J Med Internet Res. 2022;24(4):e35120. doi:10.2196/35120 35471414 PMC9092233

[26] Hellberg S, Johansson P. eHealth strategies and platforms – The issue of health equity in Sweden. Health Policy Technol. 2017;6(1):26-32. doi:10.1016/j.hlpt.2016.09.002

[27] Barreto LP, Neto FM, Paiva M, de Freitas RE, Rocha SR, Monteiro BS. Gamified mobile application as a didactic strategy for pregnant women: a proposal for health education. Procedia Comput Sci. 2024;239:437-444. doi:10.1016/j.procs.2024.06.191

[28] Briones R. Harnessing the web: how e-Health and e-Health literacy impact young adults’ perceptions of online health information. Med 2 0. 2015;4(2):e5. doi:10.2196/med20.4327 PMC4713906 26721292

[29] Cloarec J, Cadieu C, Alrabie N. Tracking technologies in eHealth: revisiting the personalization-privacy paradox through the transparency-control framework. Technol Forecast Soc Change. 2024;200:123101. doi:10.1016/j.techfore.2023.123101

[30] Sharma S, Soni S, Kaushik S, et al. SwasthGarbh: a smartphone app for improving the quality of antenatal care and ameliorating maternal-fetal health. IEEE J Biomed Health Inform. 2023;27(6):2729-2738. doi:10.1109/JBHI.2022.3211426 36191117

[31] van den Heuvel JF, Groenhof TK, Veerbeek JH, et al. eHealth as the next-generation perinatal care: an overview of the literature. J Med Internet Res. 2018;20(6):e202. doi:10.2196/jmir.9262 29871855 PMC6008510

[32] Fontein-Kuipers Y, de Groot R, van Staa A. Woman-Centered care 2.0: bringing the concept into focus. Eur J Midwifery. 2018;2:5. doi:10.18332/ejm/91492 33537566 PMC7846029

[33] Auxier JN, Bender M, Hakojärvi HR, Axelin AM. Patient engagement practice within perinatal eHealth: a scoping review. Nurs Open. 2023;10(8):4971-4984. doi:10.1002/nop2.1822 37211718 PMC10333891

[34] Andreu-Pejó L, Martínez-Borba V, Osma López J, Suso-Ribera C, Crespo Delgado E. Perinatal mental e-health: what is the profile of pregnant women interested in online assessment of their emotional state?. Nurs Open. 2023;10(2):901-914. doi:10.1002/nop2.1358 36068679 PMC9834155

[35] Monasterio G, Fernández-López MJ, Valero E, Martin U, Ayala-García A. Digital inequalities in the use of eHealth services in European public health care systems: systematic review of observational studies. J Med Internet Res. 2026;28:e81841. doi:10.2196/81841 41662579 PMC12885193

[36] Espinosa M, Artieta-Pinedo I, Paz-Pascual C, Estalella I, Maquibar A. About EMAeHealth: what do women value in a maternal education app?. A sequential mixed-method study on user perspective. JMIR Preprints. Preprint posted online March 6, 2025. doi:10.2196/preprints.73537 PMC13313032 42376428

